# Editorial: The rise of postmortem imaging in forensic radiology and paleoradiology

**DOI:** 10.3389/fradi.2026.1874150

**Published:** 2026-06-09

**Authors:** Dominic Gascho, Mislav Čavka

**Affiliations:** 1 Institute of Forensic Medicine, University of Zurich, Zurich, Switzerland; 2 University Hospital Centre Zagreb, Zagreb, Croatia; 3 School of Medicine, University of Zagreb, Zagreb, Croatia

**Keywords:** forensic identification, forensic imaging, forensic radiology, paleoradiology, photon-counting CT, postmortem imaging, postmortem investigation, virtual autopsy (VIRTOPSY)

Over the past two decades, postmortem imaging has evolved into an integral component of forensic medicine and the scientific investigation of human remains. Advances in radiological technology, particularly in computed tomography (CT) and magnetic resonance imaging (MRI), have fundamentally transformed how deceased individuals, archaeological remains, and culturally significant artifacts are examined.

**Figure 1:**
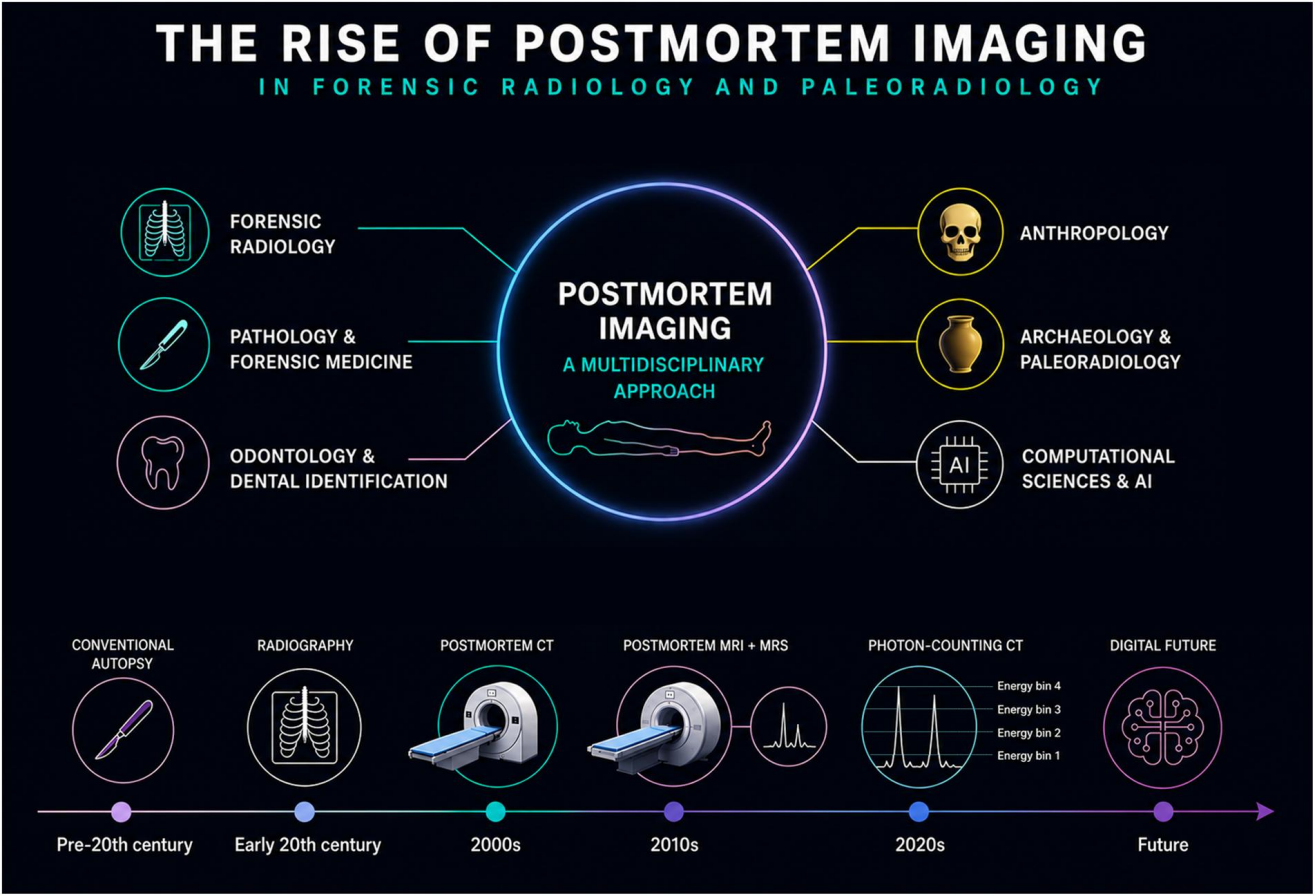
Conceptual overview of the evolution and multidisciplinary integration of postmortem imaging in forensic radiology and paleoradiology. The central diagram highlights the interdisciplinary nature of postmortem imaging, integrating forensic radiology, pathology and forensic medicine, odontology, anthropology, archaeology and paleoradiology, as well as computational sciences and artificial intelligence. The timeline illustrates the broad historical adoption and increasing use of different imaging modalities in postmortem investigations, ranging from conventional autopsy and radiography to postmortem CT, postmortem MRI/MRS, and photon-counting CT, ultimately progressing toward advanced digital and computational approaches. While postmortem CT and MRI emerged in the context of the Virtopsy concept in the early 2000s, the indicated time periods do not represent the first application of the respective technologies, but rather approximate phases during which these modalities became increasingly established in routine postmortem imaging practice. Postmortem MRI gained particular relevance from the 2010s onward, especially for pediatric and fetal investigations, whereas magnetic resonance spectroscopy (MRS) has primarily evolved as a more conceptual and research-oriented postmortem application since the late 2010s.

This Research Topic, *The Rise of Postmortem Imaging in Forensic Radiology and Paleoradiology*, brings together contributions from radiology, pathology, anthropology and odontology to reflect the maturity, diversity, and future potential of this interdisciplinary field (Figure 1).

Radiology is inherently positioned at the interface of multiple disciplines, and its application beyond clinical medicine has proven particularly impactful in postmortem contexts. In forensic medicine, virtual autopsy or Virtopsy has become an established concept describing the noninvasive or minimally invasive radiological examination of the deceased using cross sectional imaging modalities. Postmortem CT now represents a cornerstone of forensic casework in many institutions worldwide, supporting trauma analysis, detection of foreign bodies, documentation of skeletal injuries, and radiological identification. Complementary use of postmortem MRI has expanded diagnostic possibilities, particularly in pediatric cases and in the assessment of soft tissues and the central nervous system. In addition, magnetic resonance spectroscopy provides a valuable tool for non-invasive biochemical analysis.

Individual contributions in this Research Topic address the technological foundations and ongoing evolution of postmortem imaging. The article entitled *VIRTual autOPSY—applying CT and MRI for modern forensic death investigations* by Gascho outlines the application of CT and MRI as core modalities for modern forensic death investigations, emphasizing their complementary roles and current limitations while situating virtual autopsy within contemporary medico-legal practice. In the related contribution *Photon-counting CT for forensic death investigations—a glance into the future of virtual autopsy*, Gascho explores the potential of photon counting CT for forensic death investigations, demonstrating how advances in detector technology, spatial resolution, and spectral imaging may shift postmortem CT from a macroscopic toward a mesoscopic level of analysis. These developments exemplify how forensic radiology directly benefits from innovations originating in clinical radiology, while simultaneously introducing new challenges related to data volume, interpretation, and infrastructure requirements.

This Research Topic also addresses broader conceptual questions concerning the role of postmortem imaging within modern death investigation systems. Solomon et al. provide a historical and systemic perspective on the decline of conventional autopsy entitled *The evolution of postmortem investigation: a historical perspective on autopsy's decline and imaging's role in its revival*. This article highlights the role of postmortem imaging in mitigating workforce shortages, improving diagnostic documentation, and supporting public health and medico-legal objectives. Their contribution places forensic radiology within a wider societal and institutional context and reinforces the notion that postmortem imaging should be understood as a complementary component of a multimodal investigative framework rather than a universal replacement for autopsy.

An important theme emerging from this collection is the expansion of imaging-based identification techniques. While radiological identification traditionally relies on skeletal features, dental structures, and implanted devices, innovative approaches continue to broaden the forensic toolbox. The article entitled *The oral fingerprint: rapid 3D comparison of palatal rugae for forensic identification* by Petersen et al. presents an automated 3D method for comparing palatal rugae using intraoral scans, demonstrating how high resolution 3D data and computational analysis can support disaster victim identification. Although this work lies somewhat outside the classical scope of postmortem radiology, it exemplifies the increasing integration of imaging, digital modeling, and automation in forensic identification and highlights the potential for synergy between forensic radiology and forensic odontology.

Paleoradiology further extends postmortem imaging beyond medico-legal investigation into archaeology and anthropology, enabling non-destructive examination of mummified remains, historical disease patterns, and ancient mortuary practices while preserving culturally sensitive specimens.

The practical use and global dimensions of postmortem imaging are on the rise. As this field continues to expand, the implementation of postmortem CT and advanced imaging techniques remains uneven across regions, influenced by legal frameworks, resource availability, and access to specialized training. Diagnostic accuracy and interpretative reliability depend not only on imaging technology but also on professional experience, standardized protocols, and interdisciplinary collaboration. The articles in this Research Topic underscore the need for structured education in postmortem imaging for both radiologists and forensic specialists, as well as harmonized reporting practices. Furthermore, this Research Topic highlights the value of postmortem imaging for long term data preservation and scientific synthesis. Digital imaging datasets provide enduring documentation that can be revisited for second opinions, education, and research, and they enable retrospective analyses and inter institutional comparisons. In forensic medicine, archived imaging data contribute to legal proceedings, training, and methodological validation. In archaeology and anthropology, nondestructive imaging supports ethical stewardship by preserving fragile or unique remains while allowing internal assessment of skeletal material and mummified bodies.

In summary, the contributions collected in this Research Topic demonstrate that postmortem imaging has developed into a dynamic and interdisciplinary field that extends well beyond its early applications. Virtopsy and paleoradiology are increasingly interconnected domains, driven by technological innovation, societal needs, and ethical considerations. By explicitly linking methodological advances, clinical applications, historical perspectives, and emerging identification techniques, this Research Topic aims to promote collaboration and to contribute to the continued development and global dissemination of postmortem imaging as a cornerstone of modern forensic and archaeological science.

